# No effect of passive integrated transponder tagging method on survival or body condition in a northern population of Black‐capped Chickadees (*Poecile atricapillus*)

**DOI:** 10.1002/ece3.7783

**Published:** 2021-06-20

**Authors:** Jonathan J. Farr, Elène Haave‐Audet, Peter R. Thompson, Kimberley J. Mathot

**Affiliations:** ^1^ Department of Biological Sciences University of Alberta Edmonton AB Canada; ^2^ Canada Research Chair in Integrative Ecology Edmonton AB Canada

**Keywords:** Aves, individual identification, leg band, lethal effects, subcutaneous implant, sublethal effects, survival modeling

## Abstract

Passive integrated transponder (PIT) tags allow a range of individual‐level data to be collected passively and have become a commonly used technology in many avian studies. Although the potential adverse effects of PIT tags have been evaluated in several species, explicit investigations of their impacts on small (<12 g) birds are limited. This is important, because it is reasonable to expect that smaller birds could be impacted more strongly by application of PIT tags. In this study, we individually marked Black‐capped Chickadees (*Poecile atricapillus*), a small (circa 10 g) passerine, at the University of Alberta Botanic Garden to evaluate potential lethal and sublethal effects of two PIT tagging methods: attachment to leg bands or subcutaneous implantation. We used a Cox proportional hazards model to compare the apparent survival of chickadees with leg band (*N* = 79) and implanted PIT tags (*N* = 77) compared with control birds that received no PIT tags (*N* = 76) over the subsequent 2 years based on mist net recaptures. We used radio‐frequency identification (RFID) redetections of leg band PIT tags to evaluate sex‐specific survival and increase the accuracy of our survival estimates. We also used a generalized linear regression model to compare the body condition of birds recaptured after overwintering with leg band PIT tags, implanted PIT tags, or neither. Our analysis found no evidence for adverse effects of either PIT tagging method on survival or body condition. While we recommend carefully monitoring study animals and evaluating the efficacy of different PIT tagging methods, we have shown that both leg band and subcutaneously implanted PIT tags ethical means of obtaining individualized information in a small passerine.

## Graphical abstract

We investigated lethal (mortality) and nonlethal (body condition) effects of different two PIT tagging methods (implants or leg band embedded) compared with birds that were color banded but not fitted with PIT tags. In a sample of over 200 birds followed for more than two years, we found no evidence of differences in survival or body condition across any of these three treatments.

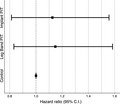

## 1 INTRODUCTION

Individual identification of focal organisms is needed to answer a range of questions in animal biology, and a variety of marking techniques have been developed to this end. Over the last two decades, the use of passive integrated transponder (PIT) tags has increased dramatically (Bridge et al., 2019). In ornithological research, radio‐frequency identification (RFID) devices are often placed at discreet locations, such as feeders or nest boxes, that PIT‐tagged birds will repeatedly visit (Johnson et al., 2013; Lajoie et al., 2019). PIT tags and RFID devices have enabled a variety of research questions to be tackled on topics including foraging behavior (Lajoie et al., 2019; Moiron et al., 2018), movement ecology (Bailey et al., 2018; Matechou et al., 2015), social networks (Brandl et al., 2021; Evans et al., 2018; Firth et al., 2015), reproductive ecology (Schlicht & Kempenaers, 2015; Schuett et al., 2012; Zangmeister et al., 2009), and physiology (Skold‐Chiriac et al., 2015; Whitfield et al., 2015). Given the range of questions this technology can be used to address, the use of PIT tags is likely to continue to grow. Evaluating the potential impacts of these devices, in terms of both lethal (e.g., survival) and nonlethal impacts (e.g., body condition, injury), is necessary to guide decisions about the range of species in which this technology can be used safely and effectively.

When considering the potential impacts of PIT tags on birds, it is important to recognize that PIT tags can be deployed using various methods which have different costs and benefits. There are two common deployment methods for PIT tags in small passerine birds: attachment to leg bands or subcutaneous implantation. Leg band PIT tags can be either affixed to leg bands with glue (Schroeder et al., 2011) or purchased embedded in colored plastic leg bands (Bailey et al., 2018; Evans et al., 2018). The advantages of leg band‐embedded PIT tags are as follows: (a) Achieving optimal arrangement and detection of tags by RFID antennae are easier with external than internal tags (Bonter & Bridge, 2011); (b) leg banding is a less invasive deployment method than internal implantation (Boisvert & Sherry, 2000); and (c) attachment of leg bands is logistically simpler than implanting PIT tags because it is faster and can be done by a single trained bird bander compared with implanting, which requires two trained bird handlers. The reported disadvantages of leg band PIT tags are that in some species, they have poorer retention than internal tags (Ratnayake et al., 2014; Schroeder et al., 2011), and in Black‐capped Chickadees, leg bands with embedded PIT tags have reportedly caused leg injuries in 1 out of 80 (~1.3%) recaptured individuals (Julie Morand‐Ferron, *personal communication*). Subcutaneous implantation uses a sterile needle to implant the PIT tag under the skin of the bird. PIT tags can be implanted between the scapulae (Nicolaus et al., 2008) or interperitoneally (Whitfield et al., 2015), but interperitoneal PIT tags have been reported to cause injuries in small passerines (Oswald et al., 2018). After implantation, sites should be sealed with adhesive glue to reduce the likelihood of tag loss (Nicolaus et al., 2008). The advantages of implants over external PIT tags are that temperature‐sensitive PIT tags can measure internal body temperature (Whitfield et al., 2015), and some studies suggest that tag loss or damage is less likely compared with externally attached PIT tags (Johnsen et al., 2005; Schroeder et al., 2011). However, confirming tag loss or migration of implanted tags is difficult without recapturing individuals (Oswald et al., 2018).

When choosing a PIT tag deployment method, it is important to consider animal welfare and minimize data bias by assessing how the specific study population may be affected by carrying PIT tags. Several studies have evaluated the effects of PIT tags on small passerine birds and found no evidence for any adverse effects (Table 1). Results such as these have led to a widespread adoption of PIT tags for field studies in small birds, including Black‐capped Chickadees (circa 10–12 g) (Bailey et al., 2018; Evans et al., 2018; Lajoie et al., 2019) and even hummingbirds (<4 g) (Hou et al., 2015). However, while the study in hummingbirds demonstrated that they could be tracked effectively with PIT tags, the study did not compare survival or body condition relative to birds that did not receive implants (Hou et al., 2015). In fact, the only other small bird (<12 g) for which the effects of PIT tags have been explicitly tested is blue tits (*Cyanistes* *caeruleus*), and this study only compared short‐term responses (up to 6 hr postimplant), and with only five individuals in the control and treatment groups (Schlicht & Kempenaers, 2018). Thus, more work is needed to explicitly test whether PIT tags have adverse impacts in small birds relative to other methods available for individual identification (e.g., color banding).

**TABLE 1:** Overview of studies investigating the effects of PIT tagging treatments on various species of small passerine birds

Species	Location	Body mass (g)	Sample size	Study subjects	Study duration	PIT tagging method	Reported effect	Reference
Great Tit (*Parus major)*	Lauwersmeer, The Netherlands	17–19	571 control, 1,339 implants	Nestlings, young adults, and adults of both sexes	2 years	Subcutaneous implantation	No effect on survival, fledgling recruitment, or body mass	Nicolaus et al. (2008)
House Sparrow (*Passer domesticus*)	Lundy Island, England	24–38	55 control, 134 implants, 55 leg bands	Adults	8 years	Subcutaneous implantation or attached to leg bands	No effect on body mass or fitness of either treatment	Schroeder et al. (2011)
Blue Tit (*Cyanistes caeruleus*)	Bavaria, Germany	10	5 control, 5 implants	Adults	6 hr	Subcutaneous implantation	Acute stress response, no long‐term effects	Schlicht and Kempenaers (2018)
Dark‐eyed Junco (*Junco hyemalis*)	Virginia, United States	18–30	215 control, 57 implants	Adults	17–19 days	Subcutaneous implantation	No effect on homing speed or return rate	Keiser et al. (2005)
Pied Flycatcher (*Ficedula hypoleuca*)	Kauhava, Finland	12–13	30 control, 278 implants	Adults	5‐ to 23‐day short term, 1‐year long term	Subcutaneous implantation	No effect on body mass or migrant survival	Ratnayake et al. (2014)
Zebra Finch (*Taeniopygia guttata)*	Western Cape, South Africa	15	21 implants	Adults	24 hr	Subcutaneous (interscapulae) and intraperitoneal implantation	Injuries and mortality reported for intraperitoneal but not for subcutaneous implants	Oswald et al. (2018)

Here, we explicitly evaluate the effect of two different PIT tagging methods on Black‐capped Chickadees in terms of both lethal and nonlethal effects relative to standard color banding. To do this, we randomly assigned chickadees to one of three treatments; control (regular banding protocol but no PIT tag), leg band (regular banding protocol including a PIT tag embedded in a color band), and implant (PIT tag implanted subcutaneously). We included regular color banding (3 color bands plus one metal band) as a control because it allowed us to compare the minimally invasive method that allows for long‐term individual identification (i.e., color bands) of resighted birds to the two PIT tag deployment methods that also allow for long‐term individual identification through resightings. Using the standard color banding method as our control treatment also allowed us to investigate a potential mechanism of leg injury for the leg bands embedded with PIT tags reported in another population of chickadees (Julie Morand‐Ferron, *personal communication*). We speculate that leg band PIT tags could be more likely to cause injuries or infections relative to normal leg bands because they are twice the height of a regular color band, which may increase the chance of dead skin or debris becoming trapped under the band and causing irritation. Additionally, mass is distributed asymmetrically on the band due to the additional mass of the PIT tag, which could lead to increased friction on the leg. Chickadees are smaller than most other nonmigratory passerines in which the effects of PIT tags have been investigated (Nicolaus et al., 2008, Schroeder et al., 2011, but see Schlicht & Kempenaers, 2018). Due to their small body size, it is conceivable that PIT tags may have a larger negative effect on chickadees relative to larger birds. Here, we present the results of a PIT tagging effort that measured the apparent survival and overwinter body condition of birds assigned to these three treatments, providing critical information to allow future studies to choose the tagging method that minimizes adverse effects.

## 2 METHODS

### 2.1 Field procedures

We conducted this study at the University of Alberta Botanic Garden in Devon, Alberta, Canada (53.4080°N, 113.7605°W). The botanic gardens are situated within the Devon Dunes natural area of the Alberta central parkland region, and native plant communities include various willow species (*Salix spp*.), balsam poplar (*Populus balsamifera*), and jack pine (*Pinus banksiana;* Natural Regions Committee, 2006). Recorded temperatures from the nearest weather station, 10 km SE at the Edmonton International Airport (YEG), range from an average daily maximum of 21.9°C during summer months (June–August) to an average daily minimum of −15°C in winter (December–March; Environment & Climate Change Canada, 2019).

**FIGURE 1:**
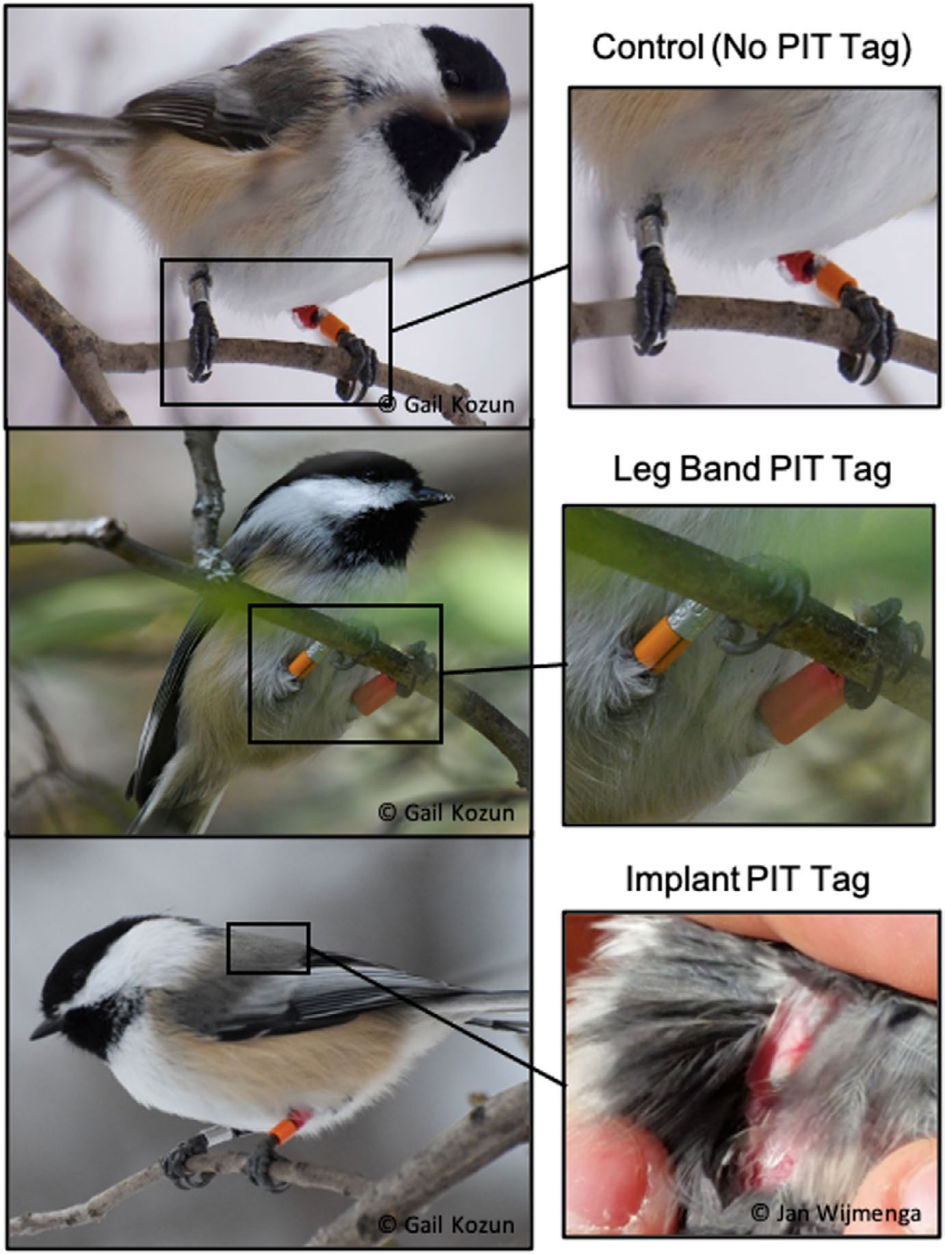
Photographs of chickadees with the three treatments: Control (no PIT tag), leg band PIT tag, and implant PIT tag. Photos by Gail Kozun and Jan Wijmenga

We used mist nets placed near feeders to capture chickadees between 15 September and 19 November 2017 (*N* = 162), and 17 September and 5 October 2018 (*N* = 73). Chickadees were banded with a Canadian Wildlife Service (CWS) metal identification ring, and we recorded body mass, tarsus length, wing chord, bill length, and bill depth. As chickadees are monomorphic, we took a small (<100 μl) blood sample from the brachial vein to determine sex molecularly (Griffiths et al., 1998). These data allowed us to verify whether our preassigned treatments were balanced with respect to body mass and sex, two variables with the potential to affect survival in chickadees (Brittingham & Temple, 1988; Desrochers et al., 1988). Dominance rank can also affect survival in chickadees (Desrochers et al., 1988; Otter, 2007), but we lack dominance rank data for our population and assume chickadees of various dominance ranks were assigned to each treatment. Chickadees were also subjected to a 2‐min novel‐environment behavioral assay as part of another study. Following this assay, chickadees were fitted with unique color band combinations for field identification, with 2 bands for chickadees with leg band PIT tags (1 regular, and 1 double height leg band with embedded RFID), and three normal color bands for control and implant birds (Figure 1).

We then fitted each chickadee with a preassigned PIT treatment: control (*N* = 76), leg band PIT tag (*N* = 79), or implanted PIT tag (*N* = 77). PIT tags are glass‐encased microchips that transmit a unique 10‐digit alphanumeric identifier when they enter the electromagnetic field of a radio‐frequency identification (RFID) antenna. The detection range of PIT tags is restricted to a maximum of 1 m from the RFID antennae depending on the size of the PIT tag. When detected at an RFID antennae, the PIT tag code and detection time are recorded, allowing for the automated collection of large amounts of data. The control treatment consisted of birds without PIT tags that were otherwise processed in the same way as PIT‐tagged birds, including receiving a unique combination of color bands. If captured birds demonstrated high levels of stress or implanting was unsuccessful, we deviated from the preassigned treatment and released the bird without a PIT tag and excluded them from all analyses (*N* = 3). Leg band PIT tags were 10 × 2 mm tags embedded in 2.6 mm colored plastic leg bands as provided by the manufacturer (Eccel Technology Ltd., Leicester, UK) and were attached following standard color banding procedure (Sutherland et al., 2004). These were determined to have a detection radius of approximately 10 cm. Implanted PIT tags consisted of 8 × 2 mm PIT tags implanted subcutaneously above the scapulae with a sterile needle (Eccel Technology Ltd., Leicester), following the implant procedure used by Nicolaus et al. (2008) on Great Tits. We used 3 M Vetbond Tissue Adhesive to seal the opening to minimize tag loss (Nicolaus et al., 2008). The smaller tag size for implants resulted in a lower detection radius (<1 cm, see below). We did not attempt to standardize handling time across our three treatments, as our aim was not to tease apart the specific mechanism of the effect of one treatment versus another (e.g., handling time versus presence of a PIT tag), but to compare treatments as they would normally be carried out in ornithological research.

### 2.2 Statistical analyses

First, we compared the sex ratios assigned to each treatment using a chi‐square test. Individuals that we were unable to assign a sex (e.g., due to failure to collect a blood sample or to adequately amplify DNA) were excluded from all analyses involving sex (*N* = 12). We found support for differences in sex ratios across treatment categories (see results, below). However, because we found no evidence for sex‐related differences in survival based on the RFID data (see below), we assumed that sex ratios across treatments did not contribute to treatment‐related differences in survival.

To further evaluate whether there were biases in treatment assignment that could influence survival and/or sublethal estimates of PIT tag effects, we performed a one‐way ANOVA to test for treatment‐related differences in body mass prior to PIT tagging. To correct for the observed differences in sex ratios across treatments, we used within‐sex centered body mass (Dingemanse & Dochtermann, 2013; Enders & Tofighi, 2007). Although chickadees show marked variation in body mass (>10% within individual variation) as a function of both ambient temperature and time of day (Brittingham & Temple, 1988), there were no treatment‐related differences in time of day or mean daily temperature at the time of capture (Table 2), and consequently, we did not correct for these variables when assessing treatment‐related differences in body mass effects.

**TABLE 2:** Results of linear regression models examining differences across randomly assigned PIT treatments in the time of day (negative reciprocal squared) and the daily mean temperature. Model fit was low for both models (time of day *R* ^2^ = 0.00017, daily mean temperature *R* ^2^ = 0.000193), indicating there were no treatment‐related differences

Effect	Estimate ± *SE*	*t*	*p*
*Time of day*			
(Intercept)	0.49096 ± 0.007768	63.204	<2 × 10^–16^*
Leg band PIT	0.000671 ± 0.0006711	0.062	.951
Implant PIT	0.002216 ± 0.01099	0.202	.840
*Daily mean temperature*			
(Intercept)	0.5267 ± 0.6912	0.762	.447
Time of day	0.1974 ± 0.9748	0.203	.840
Daily mean temperature	0.1756 ± 0.9834	0.179	.858

Note

Significant effects are indicated with an asterisk.

#### 2.2.1 Survival effects

We used a Cox proportional hazards regression model to assess differences in the survival of chickadees across control, leg band PIT, and implant PIT treatments based on recapture data from mist netting (Cox, 1972). Mist netting occurred during fall 2017 (15 September–19 November, *N* = 17 days), spring 2018 (16 January–11 March, *N* = 15 days), fall 2018 (17 September–19 November, *N* = 19 days), spring 2019 (11 March–21 March, *N* = 8 days), fall 2019 (23 October–8 November, *N* = 8 days), and fall 2020 (14 November–6 December, *N* = 8 days). Based on the first season during which each chickadee was not recaptured, we assigned it a time of death (0.5, 1, 1.5, 2, or 3 years). Time of death was assigned with the assumption that chickadees had died before the catching season after which they were not recaptured. If a chickadee was recaptured during the final catching season (fall 2020), it was right‐censored (Cox, 1972), as the time of death could not be observed. To assess the potential lethal effects of PIT tags, we compared the relative survival estimates of chickadees with leg band and implant PIT treatments to the reference category of control birds (no PIT tag). We also calculated the point estimate required to detect differences in survival of PIT tag treatments relative to control birds based on our sample sizes, assuming standard error remained constant over time.

We used PIT tag redetections of leg band chickadees at RFID equipped feeders to generate more accurate estimates of apparent survival than mist net recaptures. Although survival from RFID redetections may still underestimate actual survival due to permanent dispersal, it provides a better estimate than mist net recaptures. Only chickadees with leg band PIT tags could be reliably redetected by RFID feeder devices, due to the RFID antenna being unable to detect the smaller implanted PIT tags (Arteaga‐Torres et al., 2020) and control birds lacking PIT tags. RFID equipment became operational in the fall of 2018, and we examined apparent survival based on RFID redetections during spring 2019 (10 March–4 April), fall 2019 (18 September–21 November), spring 2020 (1 March–20 March), and fall 2020 (29 October–31 December). We truncated RFID data to avoid determining survival before 1.5 years after PIT tagging, to avoid unequal redetection effort between birds tagged in the fall of 2017 compared with the fall of 2018. We used a Cox proportional hazards model to evaluate the difference in survival between sexes using the RFID data, as sex‐related differences in survival have been reported previously for chickadees (Desrochers et al., 1988). We excluded leg banded birds with unknown sex (*N* = 2). We found no support for sex‐related differences in survival in our population (see below). Based on this, we did not account for sex in our analyses of treatment effects on survival from recapture data. We also used the RFID data to determine the extent to which our survival model based on mist netting underestimated survival. We constructed a Kaplan–Meier survival estimation comparing leg band chickadee survival based on mist net recaptures to their survival determined by RFID redetections 2 years after PIT tagging and evaluated significance with a log‐rank test. We used this analysis to estimate the degree to which the mist net recapture data overestimated chickadee mortality. We then used this factor (the ratio between the estimated probability of mortality of mist net recaptures and RFID redetections after 2 years) to correct our estimates of mortality probability for the treatment groups for which there was not RFID data.

#### 2.2.2 Body condition effects

To assess potential sublethal effects of PIT tags on chickadees, we used linear regression to compare the body mass of control, leg band, and implanted birds measured upon recapture 0.5 years after treatment assignment. We used the 0.5‐year time frame because we felt that short‐term time frames were appropriate for evaluating sublethal effects, and because using the shortest time window between successive capture sessions provided the maximum sample size. If birds with leg band or implanted PIT tags had lower body mass than control birds, this would indicate a lower body condition after overwintering with a tag (Brodin, 2007; Ratnayake et al., 2014). This analysis required repeated‐measures data and was conducted for a total sample of 68 chickadees (20 control, 24 leg band, 24 implant) captured either in the fall of 2017 or 2018 and recaptured the following spring (2018 and 2019, respectively). We used a multiple linear regression with recapture mass as a function of treatment. Sex ratios were dissimilar across treatments (proportion males: control = 0.35, leg band PIT = 0.63, implant PIT = 0.54), and chickadees exhibit sexual size dimorphism (Desrochers, 1990), which could result in differential sublethal effects based on size. Consequently, we ran models for males (*N* = 35) and females (*N* = 33) separately. We included the mass prior to PIT tagging as a covariate to control for initial differences in mass and the catching session of PIT tagging (fall 2017 or fall 2018). By including body mass at initial capture as a covariate, the effect of treatment could be interpreted as the relative change in body mass.

All statistical analyses were carried out in the R Statistical Environment (R Core Team, 2019) using RStudio (RStudio Team, 2021). We used the *survival* package for survival analyses (Therneau, 2020) and evaluated support for differences in survival based on whether the 95% confidence intervals of PIT treatments overlapped with the baseline hazard ratio of 1, which represents the survival of control birds. Nonoverlapping estimates were interpreted as showing strong support for differences.

## 3 RESULTS

There was some evidence that the sex ratios of chickadees differed across treatments (*χ* ^2^_2_ = 3.66, *p* = .16, *N* = 221). Although this did not reach conventional levels of statistical significance, the differences were potentially biologically important: The proportion of males was lower for control birds (0.46) than for both treatments of PIT‐tagged birds (leg band PIT tag = 0.60, implanted PIT tag = 0.58). Consequently, we used RFID data to investigate differences in survival based on sex to avoid sex‐related differences in response to treatments confounding estimates of treatment effects (see below). There was no support for pre‐existing treatment‐related differences in the body mass of chickadees (*F*_2, 216_ = 0.818, *p* = .443). The mean within‐sex centered body mass at the time of tagging was −0.036 g for control birds, and −0.022 g and 0.057 g for leg band and implant treatments, respectively.

**FIGURE 2:**
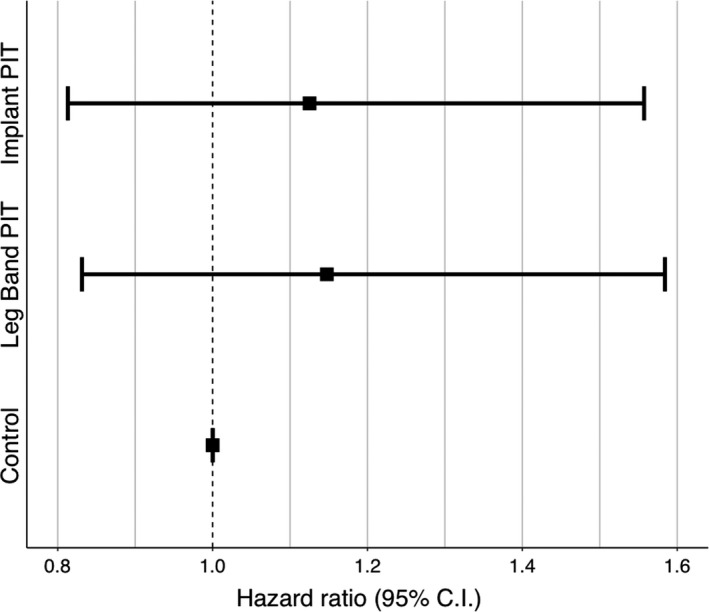
Relative survival of PIT‐tagged Black‐capped Chickadees compared with control birds using a Cox proportional hazards test. Chickadees were fitted with a preassigned treatment (control, leg band PIT, or implant PIT) in the fall of 2017 or 2018 (*N* = 232), and survival was determined based on mist net recaptures. Error bars represent 95% confidence intervals of the hazard ratio

The apparent survival of chickadees based on mist net recaptures did not differ significantly across treatments (Figure 2). Relative to control birds, leg band chickadees had a 15% higher risk of death (HR = 1.15, 95% CI [0.831, 1.584]) and a 13% reduction in survival times, while implanted birds had a 13% higher risk of death (HR = 1.13, 95% CI [0.813, 1.557]) and a 12% reduction in survival times. None of these estimates differed significantly from no effect (i.e., all confidence intervals overlapped substantially with 1).

Based on RFID redetections of leg band birds, there was no evidence of sex‐specific survival differences: Male chickadees had a 3% lower risk of death relative to females (HR = 0.97, 95% CI [0.603, 1.569]), and again, the confidence interval overlapped 1. RFID redetections also indicated that a significant proportion of surviving leg band chickadees were not recaptured in mist nets (*χ* ^2^_1_ = 52.2, *p* < .001). Two years after PIT tagging, the calculated mortality estimates were 0.949 from mist net recaptures and 0.696 from RFID redetections. This implies that mist net recaptures overestimated mortality by a factor of approximatively 1.364 (0.949 / 0.696). We used this factor to adjust the output of the Cox proportional hazards model for mist net recaptures to provide a more accurate estimate of mortality (Table 3). Performing this correction reveals that the probabilities of mortality 2 years after PIT tagging for control, leg band, and implant chickadees were 0.656, 0.696, and 0.667, respectively.

**TABLE 3:** Two‐year mortality probabilities of Black‐capped Chickadees in each treatment

Treatment	Original mortality probability^a^	Adjusted mortality probability^b^
Control	.895	.656
Leg band PIT	.949	.696
Implant PIT	.909	.667

^a^ Calculated as 1 – Kaplan–Meier survival estimates determined from mist net recaptures.

^b^ Determined based on the difference in mortality estimates between leg band mist net recaptures and RFID detections (1.364).

Body mass 0.5 years after initial capture, controlling for initial body mass, did not differ significantly across treatments for male nor female chickadees (Table 4). The mean mass ± standard deviation 0.5 years after PIT tagging did not differ for male control (11.9 ± 0.35 g, *N* = 7), leg band (11.8 ± 0.74 g, *N* = 15), and implant (11.8 ± 0.51 g, *N* = 13) nor for female control (10.8 ± 0.50 g, *N* = 13), leg band (10.9 ± 0.33 g, *N* = 9), and implant (11.1 ± 0.48 g, *N* = 11) chickadees, indicating no treatment‐related differences in the change in body condition.

**TABLE 4:** Results of two multiple linear regression models examining differences in the recapture mass of male and female chickadees across treatments^a^

Sex	Effect	Estimate ± *SE*	*t*	*p*
Male	(Intercept)^b^	2.77 ± 1.271	2.182	.0371*
	Leg band PIT	−0.0878 ± 0.155	−0.566	.575
	Implant PIT	−0.237 ± 0.160	−1.48	.150
	Mass at first capture	0.818 ± 0.110	7.41	2.99 × 10^–8^*
	Fall 2018 season	−0.586 ± 0.123	−4.75	4.75 × 10^–5^*
Female	(Intercept)^b^	2.75 ± 1.40	1.97	.059
	Leg band PIT	−0.0855 ± 0.141	−0.605	.55
	Implant PIT	0.0797 ± 0.137	0.583	.56
	Mass at first capture	0.765 ± 0.132	5.791	3.22 × 10^–6^*
	Fall 2018 season	0.106 ± 0.177	0.599	.554

^a^ Significant results are indicated with an asterisk.

^b^ Control (no PIT tag) is reference level.

## 4 DISCUSSION

A growing number of studies rely on PIT tags and RFID readers to collect a variety of individual‐level data in free‐living birds (Bonter & Bridge, 2011). Although PIT tags are used extensively in small birds, explicit tests of their impacts on birds under 12 grams are lacking. Here, we tested whether two alternative PIT tagging methods have negative effects on Black‐capped Chickadees relative to standard banding procedures. We monitored survival and body condition in chickadees for more than 2 years after they were given a control, leg band, or implant PIT tag treatment. We found no evidence for either lethal nor sublethal effect of leg band or subcutaneously implanted PIT tag treatments on chickadees relative to standard banding procedures. Survival estimates of PIT‐tagged treatments were not significantly different from control birds, and we found no differences in survival between males and females. We also show that mist net recapture data greatly underestimated apparent survival, and we adjusted survival estimates to improve accuracy using RFID redetection data. There was also no evidence of lower overwinter body condition when comparing PIT tag treatments to the control.

These findings suggest that despite small body sizes, chickadees are among the small passerine species for which PIT tags are appropriate (Keiser et al., 2005; Nicolaus et al., 2008; Ratnayake et al., 2014; Schlicht & Kempenaers, 2018; Schroeder et al., 2011). We examined survival for up to 3 years in all PIT‐tagged birds, which exceeds the average chickadee lifespan of 1.8 years (Smith, 1994). There was no evidence for deleterious effects of PIT tags during this time frame. Of the two PIT tag treatments, leg band PIT tags were detected with 100% reliability while implant PIT tags were not detected by RFID devices. The lack of detection of the implanted PIT tags was due to the manufacturer sending PIT tags with the wrong specifications and having a shorter detection radius. Unfortunately, this was not discovered until after recaptured birds were found to be alive despite not being detected at the feeders. Video recordings at the feeders that were used to evaluate the reliability of PIT tag registrations by the RFID system also confirmed that PIT implanted birds did use the feeders but were not detected (Arteaga‐Torres et al., 2020). The difference in PIT tag specifications between leg band PIT tags and implanted PIT tags precludes us from being able to do a like‐for‐like comparison of identical PIT tags deployed using different techniques (e.g., leg bands versus implants) on detectability, and we cannot assess how differences in tag orientation may also have contributed to the lower detection probability of the implanted PIT tags. However, we also observed tag loss in 31.4% of birds that received implanted PIT tags and were later recaptured and re‐examined. Tag loss, which has been previously identified by Oswald et al. (2018), occurred despite sealing implant sites with topical adhesive as advised by other studies (Becker & Wendeln, 1997; Schroeder et al., 2011).

When using RFID redetections of chickadees with leg band PIT tags, we found no meaningful differences in the sex‐specific survival, which aligns with the findings of others (Desrochers et al., 1988; Smith, 1984). Interestingly, two years after tag deployment we found that many more birds were detected by RFID (*N* = 24) than were recaptured in mist nets (*N* = 8), meaning that 66% of surviving birds would have been misidentified as “not surviving” using only mist net recapture data for our survival analyses. A potential explanation for this disparity is that previously captured birds may be exhibiting mist net aversion. Chickadees rely heavily on food caches to survive during winter, and consequently have substantial short‐ and long‐term memory capabilities (Hitchcock & Sherry, 1990; Sherry & Vaccarino, 1989). It is possible that chickadees learn to avoid repeated capture in mist nets based on visual cues from researchers setting up nets. Recapture avoidance has been reported in other small passerines following repeated exposure to mist nets, including Cliff Swallows (*Petrochelidon pyrrhonota*; Roche et al., 2013) and Pied Flycatchers (*Ficedula hypoleuca*; Camacho et al., 2017). Our survival estimates assume that recapture avoidance was equal across treatments. However, if recapture differences existed based on the PIT tagging procedure, it is conceivable that implanted birds would be the most mist net averse because of the more invasive nature of subcutaneous implantation and longer duration of handling, while leg band birds are processed almost identically to control birds and would exhibit similar levels of recapture avoidance. In this case, survival estimates for implanted birds would likely underestimate their survival relative to control birds.

While investigating potential sublethal effects of PIT tags on chickadees, we identified sex‐related differences in body mass and elevated winter body mass in our population, as previously reported in other populations of chickadees (Brittingham & Temple, 1988; Desrochers, 1990). Greater winter body mass mitigates the increased starvation risk in colder temperatures (Liknes & Swanson, 2011), and we found no evidence of changes correlated with treatment. This suggests that PIT tags did not inhibit chickadee foraging or increase energetic costs, either directly through physiological effects or indirectly by affecting social hierarchies in flocks. There is reportedly a positive correlation between body mass and dominance status (Schubert et al., 2007), because dominant birds have higher access to resources than subordinate birds (Ficken et al., 1990; Lewden et al., 2012), and the absence of differences in body mass across treatments suggests that any effects of PIT tags on chickadee social structure were negligible.

Although we found no evidence of adverse effects of PIT tagging in the current study, there are several limitations worth noting. First, given the sample sizes we had for each of the control, leg band, and implant treatment, we only had power to detect relatively large effects on survival. Assuming standard error would not change with effect size, we would be able to conclude a significant influence of leg bands or implants on survival (i.e., the lower confidence bounds would have exceeded 1) if the hazard ratios for those treatments were 1.375 and 1.379, respectively. Reassuringly, however, the point estimate probabilities of mortality 2 years following treatment after correcting for recapture probability were quantitatively very similar across all three treatments (control: 0.656, leg band: 0.696, implant: 0.667), suggesting that large treatment‐related effects on survival are unlikely. These estimates are somewhat higher compared with estimates from other chickadee populations; Brittingham and Temple (1988) identified overwinter and oversummer survival rates of 0.69 and 0.90, respectively, and Smith (1994) reported an annual survival of 0.59. When converted to 2‐year mortality estimates, this corresponds to 0.62 (Brittingham & Temple, 1988) and 0.65 (Smith, 1994). However, comparisons across studies are challenging due to differences in access to supplemental food (that inflates overwinter survival; Brittingham & Temple, 1988), habitat type (Egan & Brittingham, 1994), or recapture/resighting effort. Nonetheless, despite the very similar point estimates across our three treatment groups, we cannot exclude the possibility of small effects of PIT tags on survival, which would require much larger sample sizes to detect.

Second, we noted that leg band‐embedded PIT tags were associated with leg injuries in ~1.3% of recaptured birds in a population of urban Black‐capped Chickadees in Ottawa, Canada (Julie Morand‐Ferron, personal communication). We found no evidence of leg injuries in chickadees receiving leg band PIT tags based on visual examinations upon recapture and visual observations of leg band birds in the field, using the same make and size of leg bands as used in the Ottawa population (Eccel Technology UK, 2.3 mm bands). However, if injury rates in our population were similar to those reported previously, the sample sizes in the current study would have been expected to yield a single injured bird, which may be difficult and/or unlikely to detect. As more birds are leg banded in this population, it will be necessary to continue to monitor for potential adverse effects.

Although PIT tags are already being used extensively in many small birds (≤12 g), our study provides the most comprehensive test of the effect of PIT tags in birds of this size, both in terms of sample size and in terms of duration of follow‐up to monitor potential negative impacts. Our results lead to the reassuring conclusion that PIT tags and RFID devices can be applied to collect large amounts of minimally invasive, individual‐specific data to address a variety of questions relating to animal behavior with no detectable adverse effects relative to standard color banding. PIT tags are an effective method of identifying individuals, and the use of these technologies is predicted to continue to rise as new advancements expand their potential applications (Bonter & Bridge, 2011; Bridge et al., 2019). When adopting PIT tags, researchers should validate the use of PIT tags in their populations where possible to ensure that there are no substantial deleterious effects. In our focal population, PIT tags will be invaluable for exploring a range of future questions, as they do not compromise animal welfare and allow for the automated collection of data that accurately represents natural chickadee behavior. Based on current evidence including: (a) no support for increase in survival for implanted PIT tags relative to leg band‐embedded PIT tags, (b) the high incidence of tag loss for implanted tags, (c) no evidence of leg injuries for birds receiving leg band‐embedded PIT tags, and (d) the greater ease with which leg band‐embedded PIT tags can be applied, we suggest that leg band PIT tags may be a preferable method of PIT tag application in chickadees and other similarly sized nonmigratory birds.

## CONFLICT OF INTEREST

The authors declare that they have no conflict of interest.

## AUTHOR CONTRIBUTIONS

**Jonathan J. Farr:** Data curation (supporting); Formal analysis (lead); Visualization (lead); Writing‐original draft (lead). **Elène Haave‐Audet:** Data curation (lead); Formal analysis (supporting); Investigation (equal); Supervision (supporting); Visualization (supporting); Writing‐review & editing (equal). **Peter R. Thompson:** Formal analysis (supporting); Visualization (supporting); Writing‐review & editing (equal). **Kimberley J. Mathot:** Conceptualization (lead); Formal analysis (supporting); Funding acquisition (lead); Investigation (equal); Supervision (lead); Writing‐review & editing (equal).

### OPEN RESEARCH BADGES

This article has earned an Open Data Badge for making publicly available the digitally‐shareable data necessary to reproduce the reported results. The data is available at https://osf.io/zvfpb/.

## Data Availability

All data and R‐scripts required for the analyses presented in this study are available on the Open Science Framework Digital Repository (OSF) (https://osf.io/zvfpb/).
